# P-1056. Association of High-Risk Clones Of *P. aeruginosa* With MBLs Poses Heightened Treatment Challenge, however there is a Silver Lining

**DOI:** 10.1093/ofid/ofae631.1245

**Published:** 2025-01-29

**Authors:** Vasant Nagvekar, Neha Patel, Tejas Gohel, Shailendra Hodgar, R Srinivasan, Conrad Vas, Balaji Veeraraghavan, Anumeha gupta

**Affiliations:** Lilavati Hospital / H N Reliance Hospital, mumbai, Maharashtra, India; Hnreliance Hospital, Ahemdabad, Gujarat, India; Lilavati Hospital and Research Centre, Mumbai, Maharashtra, India; Lilavati Hospital and Research Centre, Mumbai, Maharashtra, India; Lilavati Hospital and Research Centre, Mumbai, Maharashtra, India; Lilavati Hospital and Research Centre, Mumbai, Maharashtra, India; CMCH Vellore, Vellore, Tamil Nadu, India; Lilavati Hospital and Research Centre, Mumbai, Maharashtra, India

## Abstract

**Background:**

Globally, ‘high-risk’ clones of *P. aeruginosa* continue to pose treatment challenges. These clones, in addition to intrinsic non-enzymatic β-lactam resistance mechanisms such as hyper-expressing efflux and down-regulated porins, have been acquiring broad-spectrum β-lactamases including carbapenemases. In India, recent reports indicate that most of carbapenem-resistant isolates carry MBLs in particular NDMs. However, genomic characterization of such isolates is lacking and therefore their relatedness to internationally circulating high-risk clones is not known. Herein, we have genomically characterized 35 *P. aeruginosa* index isolates collected from hospitalised patients including admitted at our intensive care unit in 2022 - 2024. We also determined their susceptibility to cefepime/zidebactam (Phase 3-stage) and other newer BL/BLIs, for both serine and MBL-producing Gram-negative bacteria.

**Figure:**
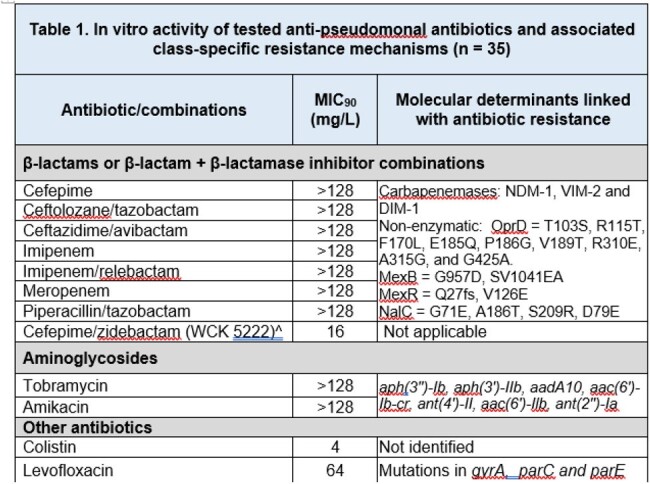

**Methods:**

The index *P. aeruginosa* pathogens were isolated from sputum, tracheal secretion, blood and drain fluid and identified by Vitek 2 (one isolate per patient). The whole genome of these isolates were sequenced (WGS) using HiSeq (Illumina 6000 platform). Susceptibilities to cefepime/zidebactam and other antibiotics were determined by reference broth micro-dilution MIC method according to CLSI M07-A11, 2018.

**Results:**

WGS revealed existence of 9 distinct multi-locus sequence types (ST), a high clonal variation despite isolates belonged to a single centre (Figure 1). Among them, 3 were international high-risk clones and nearly half of the isolated belonged to these 3 STs. Three MBL types, NDM, VIM and DIM were detected and none of the isolates carried serine carbapenemase. Mutations were noted in genes encoding efflux and impermeability. Susceptibilities to aminoglycosides and fluoroquinolones were < 30%. Except 5, all isolates were resistant to carbapenems. There was a 100% susceptibility to cefepime/zidebactam

**Conclusion:**

WGS analysis showed multiplicity of resistance mechanisms including MBLs in high-risk clones thus severely limiting treatment options. Investigational antibiotic cefepime/zidebactam may be a potential treatment option for infections caused by such isolates. In India VIM was the most common Carbapenemase resistance is being replaced by MBL(NDM)

**Disclosures:**

**All Authors**: No reported disclosures

